# Analyzing Questions About Alcohol in Pregnancy Using Web-Based Forum Topics: Qualitative Content Analysis

**DOI:** 10.2196/58056

**Published:** 2024-06-20

**Authors:** Nessie Felicia Frennesson, Julie Barnett, Youssouf Merouani, Angela Attwood, Luisa Zuccolo, Cheryl McQuire

**Affiliations:** 1 Tobacco and Alcohol Research Group School of Psychological Science University of Bristol Bristol United Kingdom; 2 Department of Psychology University of Bath Bath United Kingdom; 3 Department of Economic History School of Economics and Management Lund University Lund Sweden; 4 Health Data Science Centre Human Technopole Milan Italy; 5 Centre for Public Health Bristol Medical School University of Bristol Bristol United Kingdom

**Keywords:** social media, web-based forum, alcohol, pregnancy, prenatal health, prenatal alcohol exposure

## Abstract

**Background:**

Prenatal alcohol exposure represents a substantial public health concern as it may lead to detrimental outcomes, including pregnancy complications and fetal alcohol spectrum disorder. Although UK national guidance recommends abstaining from alcohol if pregnant or planning a pregnancy, evidence suggests that confusion remains on this topic among members of the public, and little is known about what questions people have about consumption of alcohol in pregnancy outside of health care settings.

**Objective:**

This study aims to assess what questions and topics are raised on alcohol in pregnancy on a web-based UK-based parenting forum and how these correspond to official public health guidelines with respect to 2 critical events: the implementation of the revised UK Chief Medical Officers’ (CMO) low-risk drinking guidelines (2016) and the first COVID-19 pandemic lockdown (2020).

**Methods:**

All thread starts mentioning alcohol in the “Pregnancy” forum were collected from Mumsnet for the period 2002 to 2022 and analyzed using qualitative content analysis. Descriptive statistics were used to characterize the number and proportion of thread starts for each topic over the whole study period and for the periods corresponding to the change in CMO guidance and the COVID-19 pandemic.

**Results:**

A total of 395 thread starts were analyzed, and key topics included “Asking for advice on whether it is safe to consume alcohol” or on “safe limits” and concerns about having consumed alcohol before being aware of a pregnancy. In addition, the Mumsnet thread starts included discussions and information seeking on “Research, guidelines, and official information about alcohol in pregnancy.” Topics discussed on Mumsnet regarding alcohol in pregnancy remained broadly similar between 2002 and 2022, although thread starts disclosing prenatal alcohol use were more common before the introduction of the revised CMO guidance than in later periods.

**Conclusions:**

Web-based discussions within a UK parenting forum indicated that users were often unclear on guidance and risks associated with prenatal alcohol use and that they used this platform to seek information and reassurance from peers.

## Introduction

### Background

Prenatal alcohol exposure (PAE) can lead to several detrimental outcomes, such as fetal alcohol spectrum disorder (FASD) [1] and developmental effects on both physical [2] and mental health [3]. In addition, those with FASD have a higher risk of experiencing problems in school, getting into trouble with the law, and having problems with alcohol and illicit drug use [4]. FASD has been mentioned in research since the early 1970s [5], and it is a complex diagnosis with a high rate of comorbidity [6], usually requiring a multidisciplinary team to diagnose it [7]. Estimates have shown that, on average, 9.8% of women worldwide consume alcohol in pregnancy, and in the United Kingdom, 40.1% of women report consuming alcohol in pregnancy, ranking the United Kingdom as having the fourth highest prevalence of PAE in the world [8]. It has been shown that approximately 8 in 1000 individuals have FASD in the general population globally, with the European region having a prevalence of approximately 20 in 1000, with approximately 1 in 13 children being born with FASD after PAE [9]. Estimates show that 1 in 67 pregnant women consuming alcohol gives birth to a child with fetal alcohol syndrome (the dysmorphic subtype of FASD) [8]. While there is strong evidence that high levels of PAE can be harmful, evidence on the effects of low to moderate PAE has been less conclusive. Uncertainties surrounding the risk of harm at low levels of PAE, coupled with conflicting messages from health professionals on low-moderate PAE, have been cited as reasons why some people choose not to abstain and for ongoing confusion about the risks of PAE [10]. Nevertheless, studies that have used robust methods to support causal inference have found that low-moderate levels of PAE can lead to adverse perinatal, physical [11], and developmental outcomes [12,13] and that apparent null and protective effects of PAE are likely due to residual confounding. Consequently, a recent review concluded that “any amount of prenatal alcohol exposure appeared to risk healthy child development” [14].

Previously, there was a consensus that UK guidelines on alcohol in pregnancy could be confusing because there was no clear recommendation [15]. In earlier guidelines, the National Institute for Health and Care Excellence stated that pregnant women should avoid alcohol in the first 3 months of pregnancy, and if they choose to drink, they should not drink more than 1 to 2 units twice per week [16]. In 2016, the UK Chief Medical Officers (CMOs) changed the guidelines to advise “if you are pregnant or planning a pregnancy, the safest approach is not to drink alcohol at all” [17]. This change of guidelines caused much debate. While some welcomed the introduction of the revised guidelines [18], others claimed that the new guidelines would lead to women having feelings such as guilt and anxiety if they were consuming alcohol during pregnancy [19]. Pregnant women feel there are too many guidelines to follow, arguably leading to increased stress [20]. It has also been expressed that pregnancy can lead to a perceived lack of agency and control [21] in this context, and the abstinence guideline can be perceived as “policing women” [22]. A study conducted in Denmark, a country with similar estimates of PAE as the United Kingdom, evaluated both knowledge and attitudes toward alcohol in pregnancy before and after their guidelines changed to advising women not to drink during pregnancy and showed no changes in either knowledge or attitudes [23].

Furthermore, the concept of abstaining from alcohol during pregnancy is not always clear, with some women experiencing confusion about, for example, whether food containing alcohol is safe or whether it is acceptable to consume so-called no- or low-alcohol (NoLo) products [20].

There is little research on the experiences and attitudes toward alcohol in pregnancy among the UK general population. This is particularly true for research on conversations in more informal and “naturalistic” settings, such as those on social media platforms. Within the context of the changed guidelines on alcohol in pregnancy and ongoing debates, a gap emerged in understanding informal dialogues, notably during events such as the COVID-19 pandemic. As concerns heightened regarding increased alcohol intake during pregnancy amid the lockdown, the need for research on the correlation between COVID-19 pandemic–related anxieties and evolving attitudes toward alcohol during and after the lockdown became apparent. In March 2020, the United Kingdom experienced its first lockdown [24]. This led to millions of people having to change how they lived, with everything from how to visit the midwife to having to give birth without their partners and loved ones present [25]. There was also the worry that people with alcohol dependence would not get the help they needed while the society was shut down [26]. In addition, there was a concern surrounding a potential increase in the level of alcohol consumption during pregnancy due to the stress and anxiety that the lockdown brought to many people [27]. Research has suggested that the shifts in drinking habits during the COVID-19 pandemic could have lasting effects on alcohol-related harm in the future for the general population in England [28]. However, to this date, there has been limited evidence supporting any relationship between COVID-19 concerns and increased alcohol consumption during pregnancy [29]. Therefore, more research is needed to examine the potential change in attitudes toward alcohol in pregnancy after the COVID-19 pandemic lockdown.

Many pregnant women use the internet to search for information related to their pregnancy [30]. A recent study showed that as many as 44% of new mothers used social media to keep in contact and communicate with others in the same situation [31]. Mumsnet [32] was founded in early 2000 and is one of the most prominent web-based forums for parents in the United Kingdom, with approximately 7 million monthly visitors [33]. Mumsnet was initially created as a web-based space where people could ask for and give advice and share knowledge to make parents’ lives easier [33]. While Mumsnet is open to anyone, it has previously been described as having a majority of middle-class and university-educated users [34]. Because many people use social media and the internet to seek health information [35] and to find support during pregnancy [30], Mumsnet presents an excellent opportunity for researchers to capture the unmediated opinions and thoughts about alcohol during pregnancy. The forum has previously been used to address topics such as breastfeeding [36], regretting motherhood [37], and maternal feelings [38]. Therefore, with the use of Mumsnet thread starts, this study will explore what topics related to alcohol during pregnancy are discussed and if their nature has changed since the start of Mumsnet in 2002, with the change of CMO guidelines in 2016 and the COVID-19 pandemic as key time points for comparison.

### Aims and Objectives

Given that social media and the internet can be used by those seeking to gain real-time insight into people’s behaviors and attitudes as well as to identify how people perceive public health messages [39], this study aimed to explore what issues and topics are raised with regard to alcohol use in pregnancy in web-based parenting forums. In addition, it aimed to explore if there has been a change in the different issues and topics with respect to 3 time points: before the implementation of the current CMO low-risk drinking guidelines, after the implementation of the CMO guidelines, and after the first COVID-19 pandemic lockdown.

The specific research questions in this study were as follows:

- What topics relating to alcohol in pregnancy are raised on web-based parenting forums?
- Have these topics changed in content or volume with respect to 3 time points: before the implementation of the current CMO guidelines, after the implementation of the CMO guidelines, and after the first COVID-19 pandemic lockdown?

## Methods

### Data Source

Mumsnet allows members to post anonymously on the forum called “Mumsnet Talk.” Talk consists of different subforums (eg, the “Pregnancy” subforum). Mumsnet users can post thread starts, usually by asking a question, and other users can reply to these thread starts by adding a comment. Users are identified by unique usernames. The forum is open for everyone to view; however, users must be registered to post content.

### Ethical Considerations

This study followed ethical guidelines for internet-mediated research. Ethical considerations for research using social media data differ from traditional research [40], and it is essential to distinguish between public and private data [41]. Because Mumsnet does not require users to log in to read the forum, and is available to the public, it was considered public data and informed consent was not required [42]. To support anonymity in our study, we have not included usernames and endeavored to remove personally identifiable information from the data during the cleaning process. We excluded thread starts in which the user stated that they were aged <18 years. In line with the British Psychological Society guidance [43], direct quotes were not reproduced in this study, and all quotes have been paraphrased.

A favorable ethical opinion was obtained from the School of Psychological Science Research Ethics Committee at the University of Bristol in August 2023 (ethics approval code 14455).

### Search Strategy

Data were collected through web scraping code by authors NFF and YM. The data were collected from the “Pregnancy” topic in the “Talk” part of Mumsnet and included original thread starts that mentioned alcohol in the title or text. Duplicate thread starts and threads unrelated to alcohol use during pregnancy were removed manually. After web scraping, thread starts were stored in an Excel (Microsoft Corp) file with information on the username, date and time of the thread start, and the thread start itself.

### Data Analysis

Thread starts were analyzed in 3 groups according to the time and date in which they were posted: “Pre-CMO recommendation update,” before the introduction of revised low-risk drinking guidelines in 2016 (August 24, 2002, to January 7, 2016); “Post-CMO recommendation update,” after the change of guidelines (January 8, 2016, to March 22, 2020); and “Post–COVID-19 Pandemic Lockdown,” from the first lockdown in the United Kingdom up until the last date of the data collection (March 23, 2020, to November 12, 2022). A content analysis was conducted following the steps described in the study by Elo and Kyngäs [44]. This approach was appropriate as the aim of the study was to map the landscape of discussions on alcohol in pregnancy on Mumsnet, including patterns and time trends in people’s views and experiences.

NFF read the thread starts, became familiarized with the data, and applied preliminary code labels to organize the data. An inductive approach was used because little is known about how mothers use web-based forums to discuss alcohol and pregnancy. Categories were generated after finding patterns among the codes to better understand the data [44]. Throughout the process, the categories were reviewed and refined. During each step of the analysis, both codes and categories were discussed among the researchers NFF, JB, AA, LZ, and CM and refined accordingly. We used descriptive statistics to describe the proportion of thread starts for each category for each of the prespecified periods. The analysis workflow is based on the process outlined in the study by Elo and Kyngäs [44].

## Results

### Overview

The web scraping resulted in 803 thread starts, which, after eliminating duplicates and irrelevant thread starts, resulted in 395 thread starts included in the analysis. Multimedia Appendix 1 provides an overview of the 9 categories and each code within them, together with the number of times the categories appear in each period. Although the categories are presented separately, some overlap does exist.

The results show that while the categories of Mumsnet thread starts relevant to alcohol use in pregnancy remained broadly similar over time, there were some changes in the relative prevalence of different topics over time. Category headings, frequencies over time, and illustrative quotes are presented in Multimedia Appendix 1. The categories are presented in detail in subsequent sections.

### Asking for Advice on Whether It Is Safe to Consume Alcohol or on Safe Limits

Looking at the questions raised within this category, it became apparent that the people posting on Mumsnet felt insecure about whether it is safe to consume alcohol during pregnancy or if it is safe to have a glass or 2 on a special occasion such as weddings or birthday celebrations:

I am 30 weeks pregnant and haven’t had a single drink but it is my friend’s wedding and I really want to have a glass of champagne, is this ok?

Thread starts in this category also addressed the issue of not knowing whether it is safe to eat certain foods or desserts as they contain alcohol, for example, a tiramisu or red wine sauce:

Is it ok to eat dessert that has alcohol in it? I am in my third trimester.

There was uncertainty around NoLo options, with questions raised about whether a 0.5% level of alcohol is safe to consume during pregnancy:

Is it okay to drink none alcoholic ciders? This might be a stupid question but it does say 0.5% so is there still alcohol in there that can hurt my baby?

Overall, 17.5% (69/395) of all the included thread starts appeared in this category. The category saw a slight decrease in the percentage of thread starts asking about safe limits to drink over time. Many of the thread starts in the first period, before the CMO recommendation update, mentioned the timing of the pregnancy, which could be a result of the change in guidelines.

### Consumed Alcohol Before Knowing About Pregnancy

Most of the thread starts within this category showed some expression of worry or anxiety that the thread starters had consumed alcohol before they found out about their pregnancy. They also sought reassurance from others who have been in a similar situation:

I’ve just found out I’m pregnant after weeks of unknowingly consuming alcohol and indulging in partying. I’m feeling guilty and concerned about any potential harm to my baby. Can anyone share their experiences if they have been in a similar situation?

Moreover, many thread starters mentioned that they were usually not heavy drinkers. Still, due to situations such as birthday parties or Christmas celebrations, their alcohol intake had been higher than usual:

I am freaking out please help. Just found out that I am 6 weeks pregnant and have been drinking so much, especially because of Christmas celebrations, I promise I am usually not a heavy drinker. What should I do? Could not live with myself if something happens to the baby!

Some thread starters were asking if they should consider having an abortion following an unintended PAE, even if the baby is wanted:

Need advice since I am worrying myself sick! I am pregnant and have been drinking because I was on holiday (usually only have a glass of wine once a month). Has anyone else experienced this and their baby turned out fine? Should I just have an abortion even if I really want this baby? How could I be this stupid!

Overall, 28.1% (111/395) of all the included thread starts appeared in this category. Throughout the different periods, there is a notable increase in the percentage of thread starts regarding the worry that they may have harmed their baby; this worry is expressed more frequently after the revised CMO low-risk guidelines were introduced in 2016.

### Research, Guidelines, and Official Information About Alcohol in Pregnancy

The threads started in this category were all related to research, guidelines, and information from official sources (eg, National Health Service) about alcohol in pregnancy. Thread starters throughout all periods expressed that they found this information confusing, conflicting, or untrustworthy:

It’s a bit puzzling to me. The NHS advises against it, and I’ve come across articles saying the same, yet in my real-life circle, many pregnant women I know enjoy the occasional drink, even if it’s just a glass. It got me thinking if there’s a significant gap between official recommendations and what’s happening in practice?

In the first period, thread starters were asking what others think about the guidelines and also sharing information on how it is acceptable to consume small amounts during pregnancy:

To be completely honest, there is no research showing that it is really bad for the baby!

Some skepticism toward the guidelines can be seen in how thread starters expressed that there is no evidence that small amounts of alcohol in pregnancy have an adverse effect. Those who posted in the later periods also expressed that the guidelines were not feasible for “real people” and that they were too strict while contending that most people do not follow them:

The internet just gives you information about that you shouldn’t drink and that no amount is safe but surely this is not how real people see it. I think they are just trying to scare us with all of these rules!

Of the 395 included thread starts, 46 (11.6%) of these appeared in this category. Closer to 17.5% (37/213) of the thread starts in the first period (pre-CMO updated guidance) were related to research, guidelines, and information about alcohol use in pregnancy, but this was less frequent in the later periods with 4.9% (5/103) of the thread starts in the post-CMO recommendation update and 5.1% (4/79) in the post–COVID-19 pandemic lockdown.

### NoLo Products

This category covered conversations about the consumption of NoLo products during pregnancy. Reasons for seeking NoLo alternatives included not feeling left out in a group that is consuming alcohol and also because some missed the taste of alcohol. The need for this type of product appeared greater during celebrations such as Christmas and weddings:

I don’t want to feel left out at the wedding so do you have any recommendations of what I can drink instead of champagne?

There were also thread starters who had been consuming NoLo options during pregnancy, assuming that these were completely alcohol free (ie, 0.0% alcohol by volume [ABV]) and later realized that they contained some alcohol. This was associated with a concern that this alcohol level might have harmed their babies, and they were seeking reassurance from others on Mumsnet:

I am crying so much, had a couple of none-alcohol beers and now I realised that they are 0.05%! What if I have hurt my baby?

Of the 395 included thread starts, 52 (13.2%) of these appeared in this category. All of the 3 periods included conversations about alternative products to alcohol to drink during pregnancy, and the proportions of these remained relatively stable over time.

### How to Hide Not Consuming Alcohol to Conceal Pregnancy?

This category reflected the worry thread starters felt regarding how to conceal them not consuming alcohol. This was of particular concern when they were invited to social situations where alcohol would be available:

How can I hide that I am not drinking when we go to the pub? With table service it’s going to be so much harder!

Overall, 10.4% (41/395) of all the included thread starts appeared in this category. This category saw a decrease in thread starts discussing how to hide not consuming alcohol in pregnancy during the post–COVID-19 pandemic lockdown period.

### Have Been Consuming Alcohol During Pregnancy But Now Worried

The thread starts within this category mainly address the issue of wanting reassurance that the baby will be fine although they have been consuming alcohol during pregnancy:

I am in my third trimester and had two drinks yesterday and now I am thinking that I might have hurt my baby, can someone tell me that this is fine?

Some thread starters mentioned that they have been consuming alcohol but later on read information about how alcohol can affect the baby, and therefore regret the decision to drink:

I have just had a few glasses of wine here and there and haven’t really thought of it but now I started reading about FAS and I am terrified. I cannot have an abortion since it is too late, but what should I do?

Overall, 4.3% (17/395) of all the included thread starts appeared in this category. For this category, the proportion of thread starts for the first period was 6.6% (14/213), the second period had a percentage of 1.9% (2/103), and the last period had a proportion of 1.3% (1/79), falling in this category.

### Are Consuming Alcohol During Pregnancy, Not Worried About PAE

Some of the thread starters in this category were seeking reassurance from others on Mumsnet in which they were seeking affirmation that consuming alcohol does not make them a bad person or mother:

I am going for a nice meal to celebrate, but I am worried that people will judge me if I have a drink.

Of the 395 included thread starts, 18 (4.6%) of these appeared in this category. All the thread starts in this category appeared in the first and second periods.

### Consumed Alcohol by Mistake

This category covers those who have consumed alcohol by mistake while eating a dessert, consuming nonalcoholic beers, or being served alcohol without realizing and being worried about that:

I went to the pub with my friends and ordered a non-alcohol option but after drinking most of it I realised that it was alcohol! Will I be ok? Freaking out!

There was also a concern that using other products containing alcohol, for example, a mouthwash or hand sanitizer, could have also harmed their baby:

I have been using mouthwash throughout my whole pregnancy but it has alcohol in it, what have I done? So scared right now!

Overall, 6.3% (25/395) of all the included thread starts appeared in this category. The highest percentage of thread starts in this category occurred in the post–COVID-19 pandemic lockdown period with 15% (12/79) of thread starts belonging to this category. The first period had 1.9% (4/213) of thread starts belonging to this category, and the second period had 8.7% (9/103) of the thread starts belonging to this category.

### It Is Hard Not to Consume Alcohol During Pregnancy

Although a smaller category, this category expressed a sadness or emptiness about having to give up alcohol during the pregnancy. Some users suggest that this could be a reflection of the life changes that come with having a child and also feeling left out from social situations:

I feel sad about not drinking, or maybe it’s about my life changing so much with this new baby on its way, am I the only one with these thoughts?

Overall, 4.1% (16/395) of all the included thread starts appeared in this category and the proportion of this category remained stable during the different periods.

## Discussion

### Principal Findings

This study sought to explore the topics relating to alcohol in pregnancy, which are raised on a web-based parenting forum. Moreover, it sought to explore if these topics had changed in content or volume, both after the change of CMO guidelines in 2016 (changing from advising women to abstain for the first trimester and not drinking more than 1 to 2 units per week to advising complete abstinence if pregnant or planning a pregnancy) and after the first COVID-19 pandemic lockdown in 2020. Through our content analysis of thread starts on Mumsnet, the United Kingdom’s leading parenting web-based forum, it was possible to evaluate what topics were commonly raised with regard to alcohol consumption in pregnancy and if and how these topics had changed over time. It is important to note that the 3 periods span 16, 4, and 2 years.

Much of the discussion on Mumsnet was around seeking reassurance and wanting to know if others had been in the same situation. For example, thread starters seeking reassurance that they have not harmed their babies by consuming alcohol before knowing about the pregnancy and asking if others had experienced something similar. It was evident that thread starters were not only seeking reassurance but also wanting to obtain information about alcohol in pregnancy both by asking for guidelines or wanting to know if such a thing as a safe limit exists. This brings up the risk of inaccurate information being shared among the users on Mumsnet. According to the World Health Organization [45], too much information and false information could lead to worsening outcomes in terms of health. Further research is required to investigate if this is true for the information shared on Mumsnet. In many categories, the results showed how thread starters were confused or worried about safe limits, including whether it is safe or not to consume products that may contain traces of alcohol. This is in line with previous research, showing that the concept of abstinence is not always clear, with confusion about, for example, if food containing alcohol is safe or if it is acceptable to consume nonalcohol options during pregnancy [20].

It was evident that there was a concern among the thread starters about having consumed alcohol before they found out that they were pregnant and that this caused stress and anxiety for some thread starters. There is no known safe limit for alcohol consumption during pregnancy. Some studies have shown that there was no relationship between consuming alcohol during the early days of pregnancy and outcomes such as low birth weight and spontaneous preterm birth [46], while others have suggested that alcohol consumption during the first trimester of pregnancy can increase the risk of spontaneous abortion [47,48]. Many of the thread starters had discussed the consumption of alcohol with their midwives or other professionals, but the concern remained. Previous research has shown that there is a lack of a standardized approach to how midwives approach the topic of alcohol consumption during pregnancy [16]. This could indicate that there is a need for professionals to give accurate information and at the same time being able to reduce any anxiety and stress that alcohol consumption could have caused. It has been suggested that midwives should be offered training in communication skills and in delivering alcohol interventions [16]. Reducing stress during pregnancy is especially important as it has been reported that stress can lead to outcomes such as low birth weight [49] and obesity in the offspring [50]. Research has shown that there exists a social pressure to consume alcohol, which causes a challenge when someone wishes to conceal their pregnancy [51]. This was also prevalent in the discussions on Mumsnet, where thread starters wanted advice on hiding that they were not consuming alcohol due to their pregnancy.

Our study demonstrated that the proportion of thread starts being brought up regarding alcohol consumption has changed over time. These temporal changes were most evident in the category “Research, guidelines, and official information about alcohol in pregnancy” as well as the 2 categories addressing consuming alcohol during pregnancy. The former contains topics such as not believing in the research carried out about alcohol in pregnancy or sharing information on how small amounts of alcohol are not harmful, with these all disappearing in the later periods. One topic that appeared in all periods was how confusing or conflicting the research or guidelines on alcohol consumption in pregnancy were. This is in line with previous research, showing how conflicting advice can cause stress in pregnancy and the need for reliable information [52]. Those who posted thread starts on Mumsnet were also expressing how the guidelines and research were not clear, and quotes illustrated that some felt that no “real people” could follow all the rules. This is in line with previous research about how women feel like there are too many guidelines [20] and how the abstinence message can be perceived as policing women [22]. Interestingly, thread starts asking for the guidelines or asking for more information were only observed before the introduction of the updated CMOs’ low-risk drinking guidance on alcohol in pregnancy [17]. This may indicate that the updated CMO guidance has made the recommendation to avoid alcohol during pregnancy clearer and easier to understand. The latter categories, addressing consuming alcohol during pregnancy, show that expressing that one is consuming alcohol during pregnancy on the internet was more common before the introduction of the revised CMOs’ guidance than in the later periods. This could indicate that the actual prevalence of alcohol consumption during pregnancy has gone down. However, research suggests that the prevalence still remains high [8]. It could also indicate that it has become less socially acceptable to disclose alcohol use during pregnancy. Furthermore, no threads started during the COVID-19 pandemic period expressed any alcohol consumption due to the lockdown. This is in line with previous research showing that the reported rates of alcohol consumption during pregnancy were lower after the pandemic than before the pandemic [29].

Moreover, many of the thread starts in the category “Asking for advice on whether it is safe to consume alcohol or on safe limits” during the first period mentioned the timing of the pregnancy, which could have been a result of the change in guidelines. The change in guidelines was that the previous National Institute for Health and Care Excellence guidelines suggested that pregnant women should avoid alcohol in the first 3 months of pregnancy, and if they chose to drink, they should not consume more than 1 to 2 units twice per week [16]. However, a 2020 survey by the National Organisation for FASD showed that awareness of the current CMOs guidance that the safest approach is not to consume alcohol at all if you are pregnant or if you could become pregnant remains low among some population subgroups, particularly young people (aged 18-25 years) [53]. This shows how important it is to communicate research in a way that is acceptable, understandable, and accessible for all. Our study showed that some Mumsnet users missed drinking alcohol while they were pregnant and wanted to know if others agreed that a glass of alcohol was acceptable, particularly on special occasions such as weddings or birthday celebrations. It was also evident that some of the forum users were trying to minimize the potential risks of consuming alcohol by referring to how previous generations had been consuming alcohol without clear adverse outcomes or how other countries have less strict guidelines. This was most apparent in content posted before the introduction of updated CMO low-risk drinking guidance in 2016.

The categories identified in this study highlight the importance of providing reliable and trustworthy information about alcohol consumption in pregnancy. This is relevant for scholars, professionals, and organizations, such as midwives and the Public Health of England. The study highlights the interactive nature of web-based forums, demonstrating an endeavor to establish social connections and seek peer reassurance. For future research, it is essential to investigate how these thread starts are replied to and how people manage their worries through interactions with others. It will also be important to investigate how conversations on social media can be used to identify knowledge gaps and preferences for the nature and format of prenatal health messaging and to explore the measurable impact of key public health and policy events on outcomes related to PAE.

### Strengths and Limitations

This study retrieved all available data related to alcohol in pregnancy from the largest dedicated web-based parenting forum in the United Kingdom. Moreover, to our knowledge, it is the first study to provide insight into the nature of web-based conversations on alcohol use in the United Kingdom and how the trends in these have changed over time in relation to key policy and public health events. Moreover, these findings are relevant to policy makers. This includes the current 2023 consultation [54] on NoLo products. The consultation intended to set out if the ABV that can be deemed “NoLo products” should be increased. The findings in this study suggest that increasing the threshold for ABV from 0.05% to 0.5% could exacerbate concerns among pregnant people who report having mistakenly consumed alcohol and also increase the uncertainties about the safe limits of these products. In addition, time trends in conversations are significant as they reveal uncertainties among pregnant people regarding topics such as the current CMO guidance and can offer valuable priorities to inform improved communication, reach, and preference for prenatal health information.

The choice to specifically search for mentions of “alcohol” within the “Pregnancy” topic on Mumsnet was made to ensure that only thread starts relevant to alcohol consumption during pregnancy were included, thereby excluding discussions unrelated to this specific context (such as threads discussing alcohol consumption while breastfeeding). This may have resulted in some critical thread starts being excluded. In 2021, it was reported that approximately 20,000 posts were created daily on Mumsnet [55]. Given the number of daily posts, collecting all of them and manually going through them was not feasible. For future research, natural language processing or topic modeling could be used to analyze a larger data set. Furthermore, because not everyone has the same access to the digital space, the voices heard on Mumsnet might not be representative of the United Kingdom, which could have led to some potential bias in the data. This is especially true as the demographics of Mumsnet have previously been described as middle class and university educated [34], thus omitting other socioeconomic groups from this analysis. It is important to note that previous research has shown that one predictor of alcohol consumption during pregnancy is higher education [56], which could limit the relevance of the findings to some subgroups of the general population. Previous research has also shown that social media use is more common among those in a higher socioeconomic group [57].

### Conclusions

This study provides insight into how mothers and expecting mothers use Mumsnet to raise topics that are important to them regarding alcohol consumption in pregnancy and illustrates how these topics have changed since the start of Mumsnet. The findings suggest that mothers and expecting mothers use Mumsnet primarily to seek reassurance and information from others in similar situations. Our findings also suggest that the topics and the proportion of thread starts relating to each topic have changed over time, with results indicating less confusion about the current guidelines and research about alcohol in pregnancy in more recent times. The study also provides insight into the worries and anxiety that pregnant women report experiencing if they had consumed alcohol before finding out about the pregnancy and the importance of seeking advice and reassurance from peers on how to manage that worry. These findings suggest that innovative interventions, such as peer support initiatives, may offer a promising approach to prenatal alcohol prevention, warranting further investigation.

## Appendix

Multimedia Appendix 1 Included categories.

## Abbreviations

ABV: alcohol by volume
CMO: Chief Medical Officer
FASD: fetal alcohol spectrum disorder
NoLo: no- or low-alcohol
PAE: prenatal alcohol exposure

## Data Availability

Because the site contents are copyright of Mumsnet, publishing a data set collecting posts or threads is not possible (refer to “Copyright” [58]). Instead, the summary statistics or analysis results will be published via data.bris.
