# Supplementary material for: Analyzing Questions About Alcohol in Pregnancy Using Web-Based Forum Topics: Qualitative Content Analysis
Source: JMIR Infodemiology. 2024 Jun 20;4:e58056. doi: 10.2196/58056 (PMC11224699; doi:10.2196/58056)
Supplement: Multimedia Appendix 1 [file infodemiology_v4i1e58056_app1.docx]

Multimedia Appendix 1. Included categories^a^.

| Categories | | Period | | | Paraphrased quotes illustrative of the category |
| --- | --- | --- | --- | --- | --- |
|  | | Before CMO^b^ recommendation update (n=213 thread starts) | After CMO recommendation update (n=103 thread starts) | After COVID-19 lockdown (n=79 thread starts) |  |
|  | | | | | |
| **Asking for advice on whether it is safe to consume alcohol or on safe limits, n (%)** | | 42 (19.7) | 16 (15.5) | 11 (14) | “Is it ok to eat Tiramisu? I know that it contains alcohol, but it is so good!”;  “I’m in my second trimester and it is my birthday, can I have a glass of wine to celebrate or is that stupid?”;  “I am so worried that alcohol-free isn’t really alcohol-free. Can someone please help without calling me stupid?” |
|  | Wants to know if it is okay to eat food or dessert with alcohol in it. | Yes | Yes | Yes |  |
|  | Wants to know if it is safe to use hand sanitizer or mouthwash. | Yes | —^c^ | — |  |
|  | Wants to know if it is safe to consume low or no-alcohol products. | Yes | Yes | Yes |  |
|  | Wants to know if there is a safe limit of alcohol to consume. | Yes | Yes | — |  |
|  | Wants to know if it is okay to consume alcohol because of celebrations. | Yes | Yes | — |  |
| **Consumed alcohol before knowing about pregnancy, n (%)** | | 38 (17.8) | 39 (37.9) | 34 (43) | “I am panicking, I just found out that I was pregnant and had a couple of drinks the other day. Have I hurt my baby? What if it gets [fetal alcohol syndrome] FAS? I want the baby but maybe it is better to have an abortion”;  “I cannot believe that this happened, I am pregnant and had some days of serious binge drinking (so unusual for me), I am out of my self of worry and anxiety, what should I do? Please help!” |
|  | Consumed alcohol before knowing about pregnancy, worried. | Yes | Yes | Yes |  |
|  | Consumed alcohol before knowing about pregnancy, talked to a professional, but still worried. | Yes | Yes | Yes |  |
|  | Consumed alcohol before knowing about pregnancy, mentioning termination. | Yes | Yes | Yes |  |
|  | Consumed alcohol before knowing about pregnancy, had a baby with problems. | — | Yes | — |  |
|  | Consumed alcohol before knowing about pregnancy, and had a miscarriage, wants to know if it is their fault. | — | — | Yes |  |
| **Research, guidelines, and official information about alcohol in pregnancy, n (%)** | | 37 (17.4) | 5 (4.9) | 4 (5) | “I am reading so many scary things online that I am stressing and having so much anxiety and I don’t know what to think anymore, please help.”;  “Why do different countries have different recommendations for drinking during pregnancy?”;  “What have you guys been advised about alcohol from your midwife, I feel like everyone is told different things?”;  “So what do you guys think? Back in the days everyone drank and they still do in other countries.” |
|  | Research, guidelines, and information about alcohol in pregnancy is confusing, conflicting or not trustworthy. | Yes | Yes | Yes |  |
|  | There is not enough research on alcohol in pregnancy | Yes | — | — |  |
|  | Sharing information or research to start discussion. | Yes | — | — |  |
|  | Sharing information or research on that it is okay to consume small amounts. | Yes | — | — |  |
|  | Wants to know the guidelines or more information. | Yes | — | — |  |
|  | Wants to know what others think about the guidelines. | Yes | — | — |  |
|  | There are too many restrictions. | Yes | — | — |  |
|  | Confused about guidelines vs what is feasible in real life. | — | Yes | Yes |  |
|  | There is no evidence that small amounts of alcohol have a negative effect. | — | Yes | Yes |  |
|  | Professionals gave impression that it is okay to consume alcohol. | — | — | Yes |  |
| **No- or low-alcohol (“NoLo”) products** | | 33 (15.5) | 10 (9.7) | 9 (11) | “Any ideas on drinks that are nice for Christmas whilst everyone else is drinking alcohol?”;  “I am so worried that alcohol-free isn’t really alcohol-free, can someone please help without calling me stupid?” |
|  | Giving recommendations on low or no-alcohol products. | Yes | Yes | — |  |
|  | Wants recommendations on low or no-alcohol beers. | Yes | Yes | Yes |  |
|  | Wants recommendations on low or no-alcohol drinks (for example: spirits and cocktails). | Yes | Yes | Yes |  |
|  | Wants recommendations on low or no-alcohol wine. | Yes | Yes | Yes |  |
| **How to hide not consuming alcohol to conceal pregnancy, n (%)** | | 21 (9.9) | 16 (15.5) | 4 (5) | “Still such early days but how do I hide not drinking from my friends and family? I cannot blame driving since we do not have a car. Please help as I don’t want to tell people yet!” |
|  | Wants recommendations on how to hide that they are not consuming alcohol. | Yes | Yes | Yes |  |
|  | Wants to know if consuming small amounts of alcohol to hide pregnancy is okay. | — | Yes | — |  |
|  | COVID-19 makes it harder to hide that they are not consuming alcohol. | — | — | Yes |  |
|  | COVID-19 makes it easier to hide that they are not consuming alcohol. | — | — | Yes |  |
| **Have been consuming alcohol during pregnancy but now worried, n (%)** | | 14 (6.6) | 2 (1.9) | 1 (1) | “Just had like two sips of a drink and was so judged by my friend that it made me worried, what do you guys think? Should I be worried?” |
|  | Have been consuming alcohol during pregnancy but now worried. | Yes | Yes | — |  |
|  | Have been consuming alcohol during pregnancy but now worried because of change of guidelines. | Yes | — | — |  |
|  | Consumed alcohol in secret but now worried. | — | Yes | Yes |  |
| **Are consuming alcohol during pregnancy, not worried about PAE^d^, n (%)** | | 15 (7) | 3 (2.9) | 0 (0) | “I have a glass of wine once in a while even if I know it’s not good for the baby.”;  “I am now in my second trimester and can finally start having a drink once in a while.” |
|  | Will be consuming alcohol during pregnancy. | Yes | — | — |  |
|  | Had a drink but it made them feel sick. | Yes | — | — |  |
|  | Consuming alcohol and wants reassurance that it is okay. | Yes | — | — |  |
|  | Relaxed later in pregnancy when it comes to consuming alcohol or in second pregnancy. | Yes | — | — |  |
|  | Does not feel like they are allowed to do anything. | — | Yes | — |  |
|  | Had a drink but was judged for it. | — | Yes | — |  |
| **Consumed alcohol by mistake, n (%)** | | 4 (1.9) | 9 (8.7) | 12 (15) | “I just had the most amazing dessert but then I realised that it has alcohol in it, have I done something really bad that has hurt the baby?”;  “I am crying so much, had a couple of non-alcohol beers and now I realised that they are 0.05%! What if I have hurt my baby?” |
|  | Have been consuming no- or low alcohol product and now worried. | Yes | Yes | Yes |  |
|  | Consumed alcohol by mistake and now worried. | Yes | Yes | Yes |  |
|  | Had food or dessert with alcohol and now worried. | — | Yes | Yes |  |
|  | Used hand sanitizer or mouthwash and now worried. | — | Yes | Yes |  |
|  | Wants to know if alcohol can transfer from others. | — | — | Yes |  |
| **It is hard to not consume alcohol during pregnancy, n (%)** | | 9 (4.2) | 3 (2.9) | 4 (5) | “I really want a nice cold pint in this hot weather, is anyone else struggling with not drinking?” |
|  | Miss drinking. | Yes | Yes | Yes |  |
|  | Wants to consume alcohol but not doing it. | — | — | Yes |  |

^a^Percentages report the proportion of thread starts that fall within each period for each category.

^b^CMO: Chief Medical Officer.

^c^There are no thread starts falling under the code.

^d^PAE: prenatal alcohol exposure.
